# The interferon-gamma pathway is selectively up-regulated in the liver of patients with secondary hemophagocytic lymphohistiocytosis

**DOI:** 10.1371/journal.pone.0226043

**Published:** 2019-12-17

**Authors:** Giusi Prencipe, Claudia Bracaglia, Ivan Caiello, Antonia Pascarella, Paola Francalanci, Manuela Pardeo, Alessandra Meneghel, Giorgia Martini, Marianna N. Rossi, Antonella Insalaco, Giulia Marucci, Valerio Nobili, Marco Spada, Francesco Zulian, Fabrizio De Benedetti

**Affiliations:** 1 Division of Rheumatology, Bambino Gesù Children’s Hospital, IRCCS, Rome, Italy; 2 Department of Pathology, Bambino Gesù Children’s Hospital, IRCCS, Rome, Italy; 3 Department of Woman and Child Health, University of Padua, Padua, Italy; 4 Hepatology Gastroenterology and Nutrition Disease Unit, Bambino Gesù Children's Hospital, IRCCS, Rome, Italy; 5 Division of Abdominal Transplantation and Hepatobiliopancreatic Surgery, Bambino Gesù Children's Hospital, IRCCS, Rome, Italy; Panjab University, INDIA

## Abstract

Aim of this study was to investigate the activation of the IFNγ pathway in the affected liver and in the blood of patients with secondary hemophagocytic lymphohistiocytosis (sHLH). To this purpose, the mRNA expression levels of *IFNG* and IFNγ-inducible genes as well as Tyrosine (701)-phosphorylated signal transducer and activator of transcription 1 (STAT1) protein levels were evaluated in the liver and in peripheral blood mononuclear cells (PBMCs) of three patients with sHLH with predominant liver involvement. The mRNA expression levels of *IFNG* and IFNγ-inducible genes were markedly higher in patient livers compared to control livers and to one disease control liver. Conversely, slight differences in the expression levels of Type I IFN-inducible genes and other classical inflammatory cytokine genes were found. Further supporting the activation of the IFNγ pathway, higher protein levels of phosphorylated and total STAT1 were detected in patient livers compared to control livers. When the expression of the same genes analysed in liver tissues was evaluated in PBMCs collected from 2 out of 3 patients before the liver biopsy, we found that mRNA levels of IFNγ-inducible genes were markedly increased. Accordingly, high circulating levels of IFNγ-inducible CXCL9 were observed in patients. Altogether, these data demonstrate the selective and marked up-regulation of the IFNγ pathway in the liver tissue and blood of patients with active sHLH. Finally, we show that measurement of circulating CXCL9 levels and evaluation of IFNγ–inducible gene expression levels in PBMCs may represent a new valid tool to better identify patients with suspected HLH with predominant liver involvement.

## Introduction

The term hemophagocytic lymphohistiocytosis (HLH) identifies a unique clinical life-threatening syndrome characterized by a hyperinflammatory state, caused by an overwhelming activation of T lymphocytes and macrophages. HLH are classified in primary and secondary forms [1]. Primary or familial HLH (pHLH) is caused by biallelic mutations in genes coding for proteins involved in the cytotoxic activity of T lymphocytes and Natural Killer (NK) cells. Secondary or acquired HLH (sHLH) occurs, in the absence of known genetic causes, in the context of infections, malignancies and rheumatic diseases (the latter being referred to as macrophage activation syndrome, MAS) [2]. Although primary and secondary HLH share clinical and biochemical features, their pathogenesis is different [3]. Indeed, while the etiology of pHLH is the defect in cytotoxicity of T lymphocytes and NK cells, the causes leading to sHLH are still not clearly established. Whatever the causes, a vast body of evidence from experimental models of primary and secondary HLH and observational studies in patients points to the high production of IFNγ playing a pivotal role in the hyperinflammation of all HLH forms [4–8].

Liver involvement is very common in HLH. Although not considered a formal diagnostic criterion, HLH patients almost always (90%-98%) have evidence of hepatitis ranging in severity from mild elevation of transaminases to fulminant hepatic failure. Occasionally, liver involvement may dominate the clinical presentation. Acute liver failure may be the presenting feature in HLH, particularly in neonates [9–11].

In order to obtain further insight into the molecular mechanisms involved in sHLH, in a target tissue, we investigated the activation of the IFNγ pathway in the affected liver and in PBMCs from patients with active sHLH with predominant liver involvement, by evaluating the mRNA and protein expression levels of IFNγ-regulated mediators and the activity of the transcription factor signal transducer and activator of transcription 1 (STAT1).

## Patients and methods

### Patients and sample collection

Liver tissues and blood samples were collected after parent/patient provided written informed consent. The Bambino Gesù Children's Hospital Institutional Ethical Committee approved the study (number 1658/2018).

**Fig 1:**
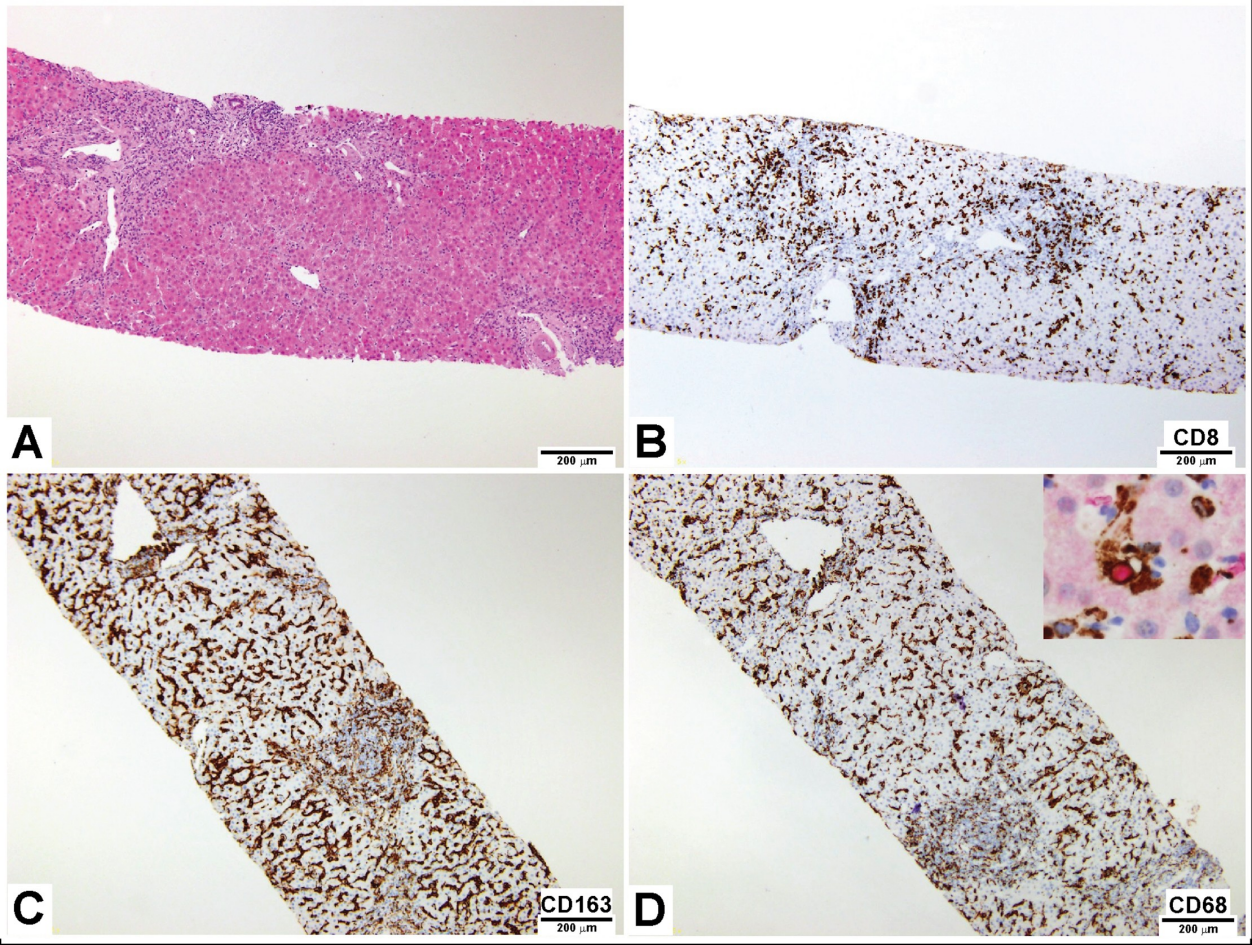
Patient 1 liver histology. **A)** Hematoxylin-eosin staining shows dense portal and sinusoidal inflammatory infiltrate, HE 10x. Anti-CD8 **(B)** displays T-lymphocytes, and anti-CD163 **(C)** and CD68 **(D)** reveal numerous macrophages within sinusoids, 10x. Double immunostaining with CD68 and Glycophorin (inset in panel D) helps to identify histiocytes engaged in hemophagocytosis, 40x.

Patient 1 (P1), a 24 years old Caucasian boy with a history of three episodes of sHLH of unknown cause (all fulfilling the HLH-2004 criteria [12]) presented, 8 years after the last episode, with progressive increase in transaminases and ferritin, while in excellent general conditions and with no fever. Cell blood counts and acute phase reactants were normal (Table 1). Bone marrow aspirate showed some activated macrophages without overt hemophagocytosis. The liver biopsy, performed before initiation of HLH treatment, exhibited massive infiltration of portal tracts and sinusoids from CD163-positive and CD68-positive macrophages, some of which displaying hemophagocytosis, and dense CD8-positive T cell infiltrates (Fig 1A–1E).

**Table 1:** Patients’ laboratory parameters.

	Patient 1			Patient 2			Patient 3		
Days from liver biopsy (day 0)	-7	-3	0	-7	-4	0	-7	-3	0
**Ferritin (ng/mL)**	1954	2220	2807	343	606	765	3253	1972	1522
**White blood cells (10^3 /uL)**	7.47	5.54	5.40	6.47	4.82	3.45	5.51	5.85	5.04
**Neutrophils (10^3 /uL)**	4.22	2.87	2.73	2.54	1.77	1.17	2.24	2.1	1.88
**Hb (gr/dl)**	15.9	16.5	16	11.5	10.7	10.3	11.8	12.5	11.8
**Platelet (x10**^**^9**^**/L)**	218	205	213	243	143	179	170	247	239
**CRP (mg/dl)**	0.41	0.39	0.56	1.16	0.83	0.73	0.29	0.29	NA
**Fibrinogen (mg/dL)**	256	236	227	310	303	320	240	270	NA
**Triglycerids (mg/dL)**	149	178	187	147	118	122	158	223	NA
**LDH (U/L)**	486	695	739	2128	5799	2713	324	231	NA
**ALT (U/L)**	1776	2298	2832	887	3996	1921	1065	613	329
**AST (U/L)**	670	965	1153	702	3273	1096	435	158	170
**GammaGT (U/L)**	209	278	290	28	130	139	103	110	103
**IFNγ (pg/ml)**	<16	<16	NA	137	230	NA	NA	NA	<16
**CXCL9 (pg/ml)**	2911	5954	NA	24836	30437	NA	NA	NA	641
**CXCL10 (pg/ml)**	841	1838	NA	8758	9799	NA	NA	NA	908
**IL-18 (pg/ml)**	787	1420	NA	3091	2915	NA	NA	NA	62000
**Liver CD163-positive cells**			+++			+			+++
**Liver CD68-positive cells**			+++			+			+++
**Liver CD8-positive cells**			++			+			+

**+** Weak staining

++ moderate staining

**+++** strong staining

NA, Not Available

Patient 2 (P2), a Caucasian boy, presented at age of 2 years with the first episode of sHLH (fulfilling the HLH-2004 criteria [12]) with severe liver involvement, associated with panniculitis and EBV infection. Sixteen months later, he developed a new episode of panniculitis without fever followed by a progressive increase in transaminases and ferritin with normal cell blood count (Table 1). At this time, he did not present an EBV infection nor reactivation. A liver biopsy, performed before initiation of HLH treatment, showed massive infiltration from CD163-positive and CD68-positive macrophages, in the absence of evidence of hemophagocytosis. Infiltrating CD8-positive T cells were also observed.

Patient 3 (P3), a Caucasian girl, presented at age of 13 years with onset of systemic juvenile idiopathic arthritis (sJIA) complicated by MAS (fulfilling the 2016 MAS classification criteria [13]). Treatment with glucocorticoids and anakinra was started with progressive improvement of clinical conditions and laboratory parameters. During glucocorticoid tapering, she developed a progressive increase in ferritin and transaminase, while cell blood count and acute phase reactants remained normal (Table 1). While on glucocorticoids (1mg/kg/day), a liver biopsy was performed, that showed infiltration from CD163-positive and CD68-positive macrophages, with no evidence of active hemophagocytosis, and from CD8-positive T lymphocytes.

Functional analysis, perforin expression, degranulation assay and NK cytotoxic activity were normal in all 3 patients. Analysis of primary HLH-related genes (*PRF1*, *UNC13D*, *STX11*, *STXBP2*, *RAB27A*, *XIAP*, *SH2D1A*) showed a heterozygous variant (R928C) in the *UNC13D* gene in P1 and was negative for P2 and P3.

As disease liver control, we used a liver biopsy specimen collected from a patient (P4) with an undefined relapsing inflammatory disorder presenting with suspected autoimmune hepatitis. The liver biopsy exhibited mild infiltration of CD68-positive and CD163-positive macrophages and of CD8-positive lymphocytes.

As control livers, three liver biopsy specimens were obtained (per protocol during liver transplantation) after graft reperfusion in paediatric liver transplantations.

### Peripheral blood mononuclear cell isolation, RNA extraction and quantitative Real Time PCR

Peripheral blood mononuclear cells (PBMCs) from patients and controls were isolated by Ficoll centrifugation (LiStarFish).

Total RNA was extracted from snap frozen percutaneous liver biopsy specimens and PBMCs using Trizol (Life technologies). cDNA was obtained using the Superscript Vilo kit (Invitrogen). Real-time PCR assays were performed using the TaqMan Universal PCR Master Mix (Applied Biosystems) and TaqMan gene-expression assays (Applied Biosystem). Gene expression data were normalized using *HPRT1* (Applied Biosystem) as endogenous control. Data are expressed as arbitrary units (AU), determined using the 2^-Δct^ method.

### Type I IFN, Type II IFN and inflammatory cytokine scores calculation

Type I IFN score was calculated as reported by Rice et al. [14]. With the same approach, we calculated a Type II IFN score by using the expression levels of 5 IFNγ-inducible genes (*CXCL9*, *CXCL10*, *CXCL11*, *IDO1* and *CIITA*) and an inflammatory cytokine score by using the expression levels of 6 inflammatory cytokine genes (*TNF*, *IL1B*, *IL18*, *IL12A*, *IL12B* and *IL10)*.

### Protein extraction and western blot analysis

Total tissue and PBMC proteins were extracted using RIPA Buffer (Cell Signalling). For western blotting, protein lysates were resolved by 10% SDS-PAGE. Proteins were then transferred to nitrocellulose membranes (Amersham Life Sciences) and probed with poly-clonal rabbit antibodies to phospho-Tyr701 STAT1, total STAT1 and GAPDH (all by Cell Signalling), by using standard procedures.

### Cytokine measurements

Plasma levels of CXCL9 were measured using the human Duoset ELISA KIT (R&D Systems Inc.). IFNγ and IL-18 plasma levels were measured using the IFNγ quantikine Elisa Kit (R&D Systems Inc.) and the IL-18 ELISA KIT (MBL International), respectively.

### Histology and immunohistochemical analysis

Liver biopsies were obtained for pathological examination. Standard morphological evaluation was based on hematoxylin and eosin (H&E) sections of formalin fixed specimens. To characterize the inflammatory infiltrates, immunohistochemical markers for macrophages (CD68, clone PG-M1, Dako, Glostrup, Denmark; CD163, clone 10D6, Thermo Scientific, Fremont, CA, USA), T lymphocytes (CD8, clone C8/144B, Dako, Glostrup, Denmark) and erythroid cells (Glycophorin, clone JC159, Dako, Glostrup, Denmark) were applied. Four-micron thick tissue sections were deparaffinised with xylene and rehydrated with graded alcohols. After washing in distilled water, sections were immersed in 3% hydrogen peroxide to block endogenous peroxidase.

## Results

### IFNγ pathway activation in liver tissue

**Fig 2:**
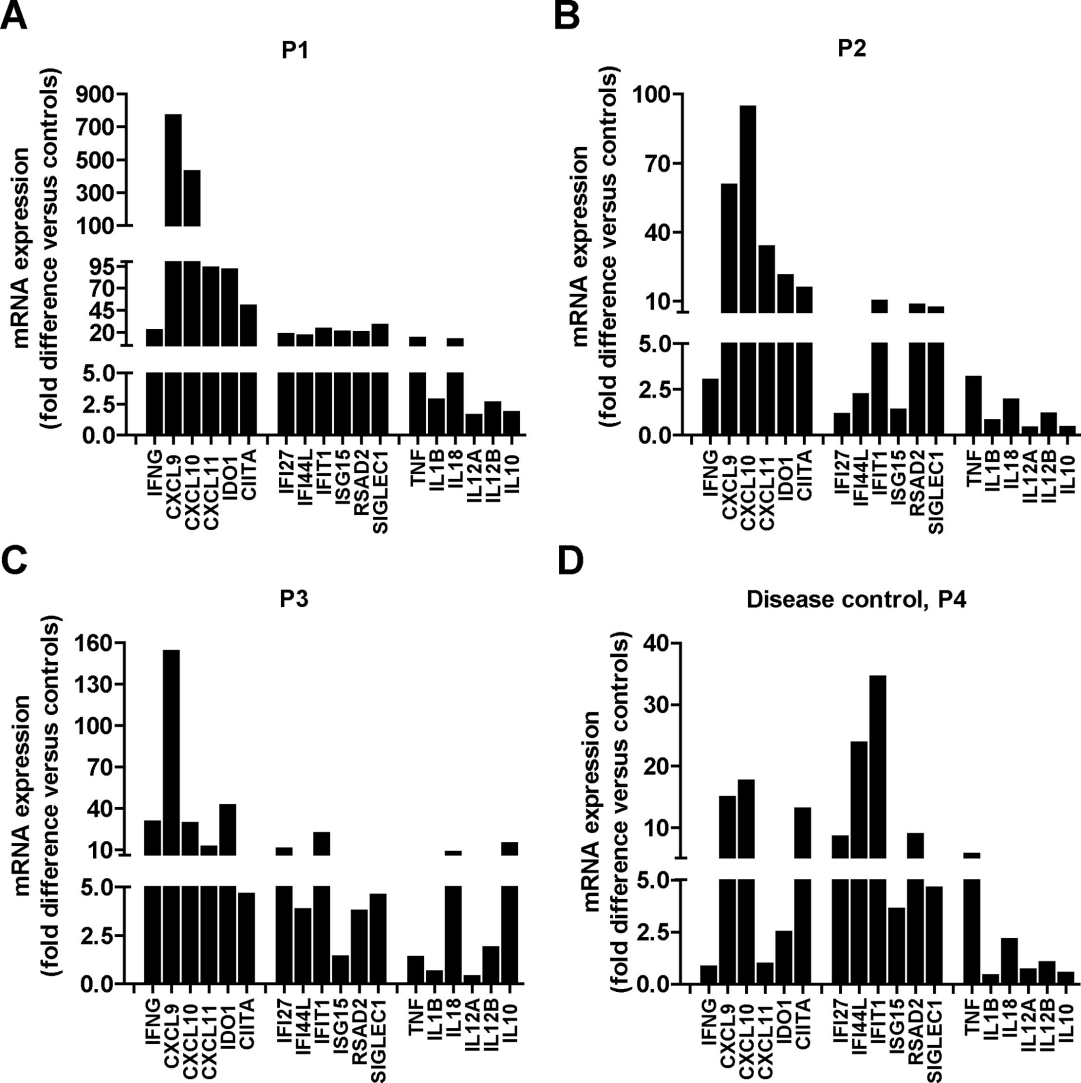
The IFNγ inducible genes are up-regulated in patient livers. mRNA expression levels of *IFNG*, IFNγ-inducible genes (*CXCL9*, *CXCL10*, *CXCL11*, *IDO1*, *CIITA*), Type I IFN-inducible genes (*IFI27L*, *IFI44L*, *IFIT1*, *ISG15*, *RSAD2*, *SIGLEC1*) and inflammatory cytokine genes (*TNF*, *IL1B*, *IL18*, *IL12A*, *IL12B*, *IL10*) were assayed in the liver from P1 **(A)**, P2 **(B)**, P3 **(C)** with sHLH and from P4 **(D)** with autoimmune hepatitis (disease control). Results are expressed as fold difference as compared with mean values obtained from control livers (n = 3), after normalization with the housekeeping gene *HPRT1*.

In order to investigate the potential pathogenic role of IFNγ in the affected liver of patients with sHLH, mRNA expression levels of *IFNG* and of the IFNγ-inducible genes (*CXCL9*, *CXCL10*, *CXCL11*, *IDO1* and *CIITA*) were analysed. mRNA expression levels of *IFNG* and IFNγ-inducible genes were strikingly higher in liver tissues collected from all patients, compared to 3 control livers (Fig 2A–2C). To determine if the up-regulation of *IFNG* and IFNγ-inducible genes was selective, mRNA expression levels of six known Type I IFN-inducible genes (*IFI27L*, *IFI44L*, *IFIT1*, *ISG15*, *RSAD2*, *SIGLEC1*) and of other classical inflammatory cytokines (*TNF*, *IL1B*, *IL18*, *IL12A*, *IL12B*, *IL10*) were evaluated. Compared to controls, livers from the 3 sHLH patients had a mild increase or a decrease in the expression levels of these genes. Notably, in the liver from P4 with autoimmune hepatitis we did not find increased *IFNG* mRNA levels (Fig 2D). Consistent with this result, we found only a mild increase in some IFNγ-inducible genes as well as of some Type I IFN-inducible genes.

**Fig 3:**
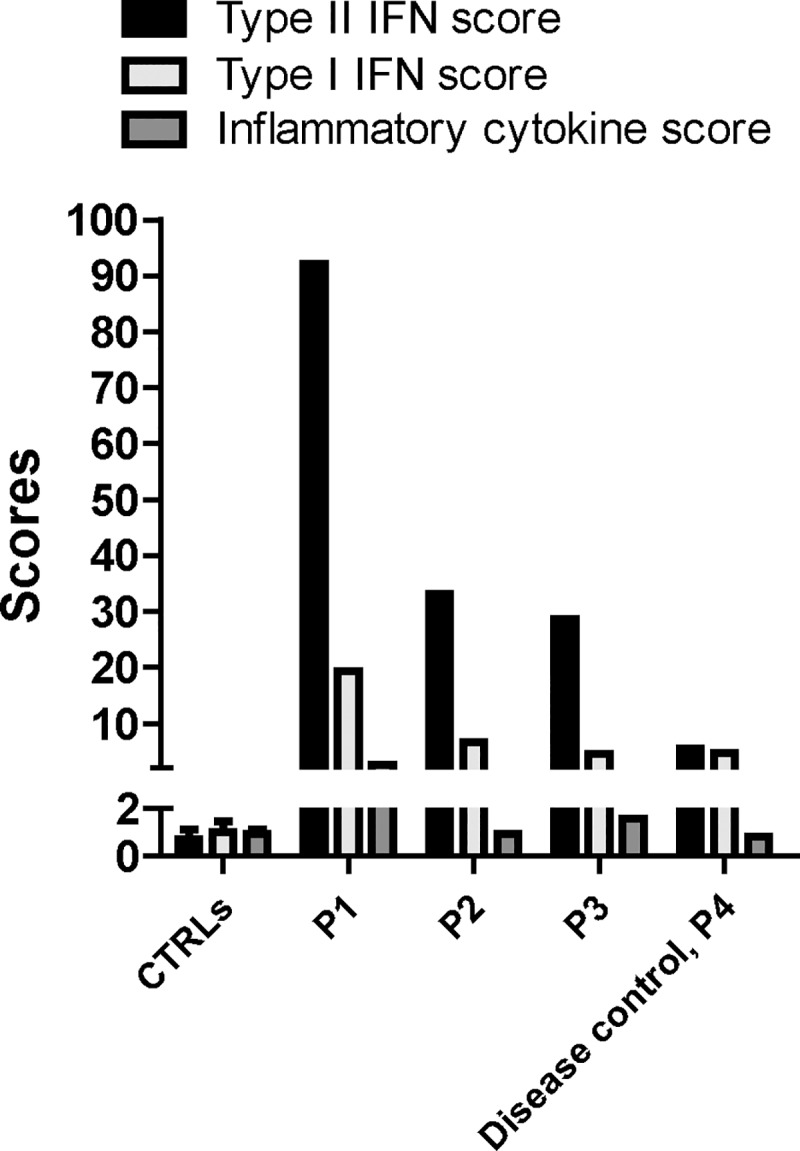
The Type II IFN score is up-regulated in sHLH patient livers. The Type II IFN score, the Type I IFN score and the inflammatory cytokine score have been calculated for P1, P2, P3 and P4 liver biopsies as described in the material and methods paragraph.

A Type I IFN score, based on the 6 genes analysed in this study, is widely accepted as a measure of the activation of the Type I IFN pathway [14, 15]. With a similar approach, we derived a Type II IFN score using the expression levels of the five IFNγ-inducible genes analysed as well as an inflammatory cytokine score using the expression levels of the six inflammatory cytokine genes analysed. We found that the Type II IFN score in P1, P2 and P3 was markedly elevated (92.2, 33.2 and 28.7, respectively) also compared to the disease control P4 (5.5), (Fig 3). In contrast, the Type I score was moderately increased and in 2 out of 3 sHLH livers (19.4, 6.6 and 4.5, respectively) it was comparable to that of the disease control P4 (4.7). Similarly, the inflammatory cytokine score was marginally increased in sHLH livers (2.7, 0.9 and 1.6, respectively) compared to that of the disease control (0.8).

**Fig 4:**
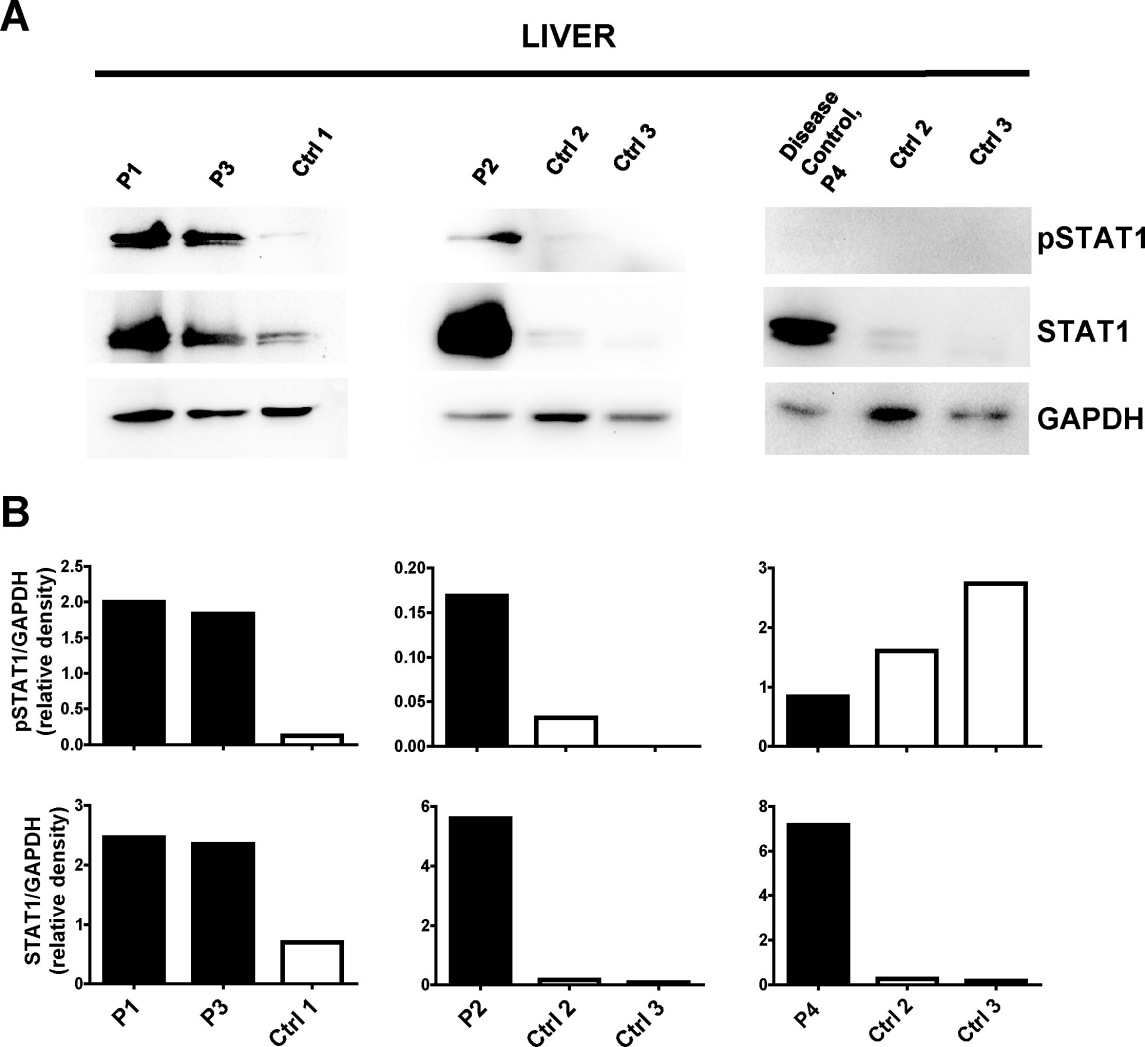
Tyrosine 701-phosphorylated STAT1 and total STAT1 levels are higher in sHLH patient liver biopsies. **A)** Tyr(701)-phosphorylated STAT1 (pSTAT1) and total STAT1 protein levels were evaluated by Western Blot analyses in patient and control total liver lysates. **B)** pSTAT1 and STAT1 relative density values were reported.

Based on our previous data obtained in a mouse model of MAS, showing that the up-regulation in the mRNA expression levels of *IFNG* and IFNγ-inducible genes in liver and spleen was associated with an increase in phosphorylated STAT1 protein levels [8], we evaluated Tyr(701)-phosphorylated STAT1 levels in liver biopsies. Tyr(701)-phosphorylation of STAT1 was markedly higher in livers from patients with sHLH than in livers from controls and from the disease control (Fig 4A and 4B). Furthermore, in agreement with the data of Hu X. et al. [16], demonstrating that IFNγ up-regulates STAT1 expression, a remarkable increase in the tissue levels of total STAT1 was also observed (Fig 4A and 4B).

Altogether, these results demonstrate that a selective activation of the IFNγ pathway is present in the liver tissue of patients with sHLH.

### IFNγ pathway activation in peripheral blood

**Fig 5:**
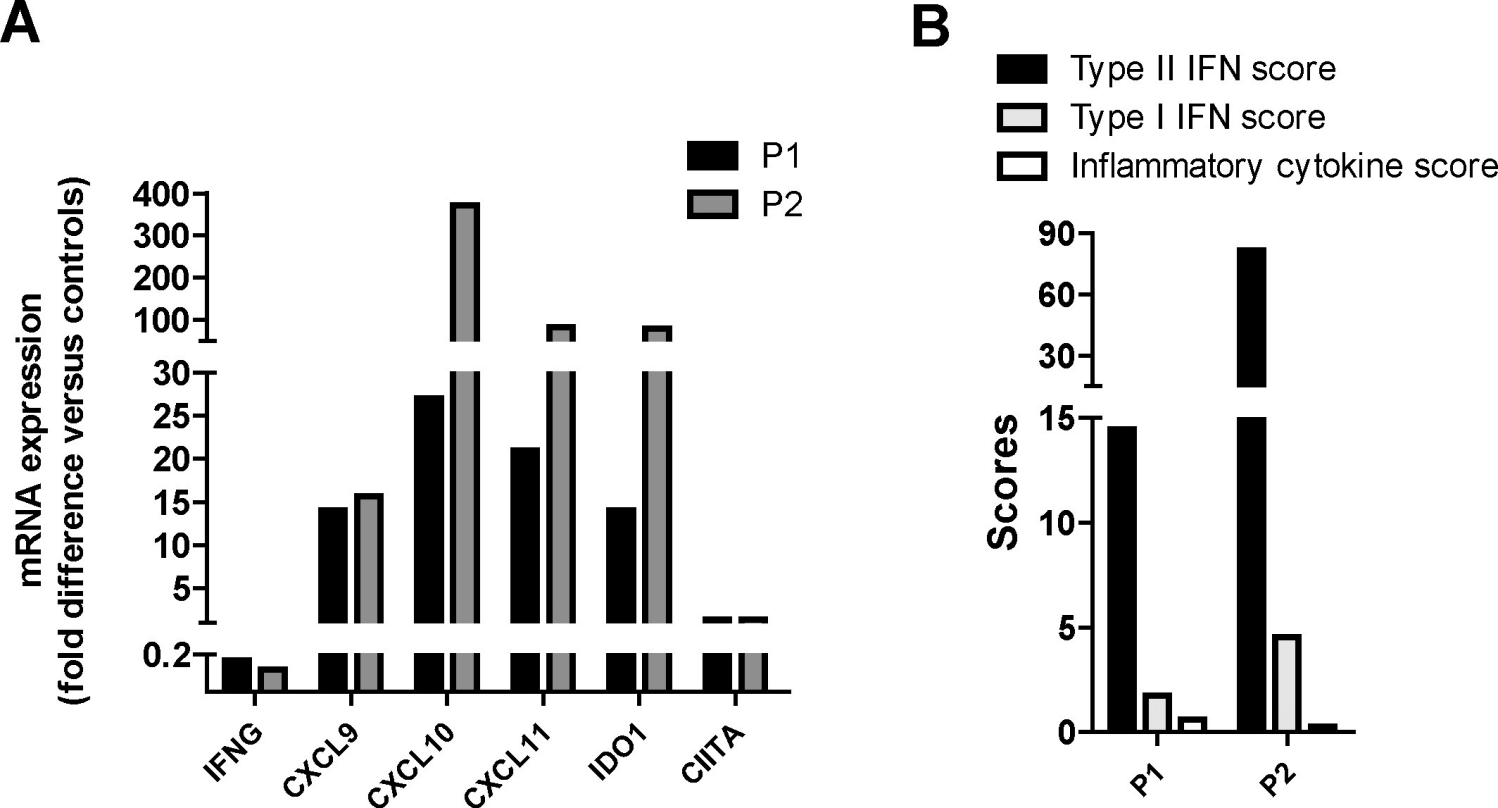
The IFNγ signature is up-regulated in PBMCs of P1 and P2 collected during the active phase of sHLH. **A)** mRNA expression levels of *IFNG* and IFNγ-inducible genes were evaluated in PBMCs collected from P1 and P2 at early stages of disease and compared to those observed in PBMCs collected from healthy controls (n = 3). Results are obtained after normalization with the housekeeping gene *HPRT1* and are reported as fold difference versus the mean values obtained in controls. **B)** Type II IFN score, Type I IFN score and inflammatory cytokine score in PBMCs were calculated as reported in materials and methods.

We then studied mRNA expression levels of *IFNG* and IFNγ-inducible genes in P1 and P2 (no sample available for P3) in PBMCs collected at early stages of the disease, before liver biopsy was performed and before treatment was initiated. In evident contrasts with the findings in liver tissues, in PBMC samples, *IFNG* mRNA expression levels were lower than those observed in healthy controls (Fig 5A), showing that overexpression of *IFNG* is limited to disease target organs and does not occur in the blood. However, when we analysed mRNA expression levels of IFNγ-inducible genes in PBMCs, we found that mRNA levels of *CXCL9*, *CXCL10*, *CXCL11*, *IDO1*, *CIITA* and, consistently, the Type II IFN score, were higher than those observed in healthy controls (Fig 5A and 5B). The Type I IFN score as well as the inflammatory cytokine score were only marginally increased (Fig 5B). Altogether, these data show that the IFNγ pathway is selectively up-regulated also in PBMCs, in addition to liver tissue, even in the absence of peripheral blood mRNA overexpression of IFNG.

**Fig 6:**
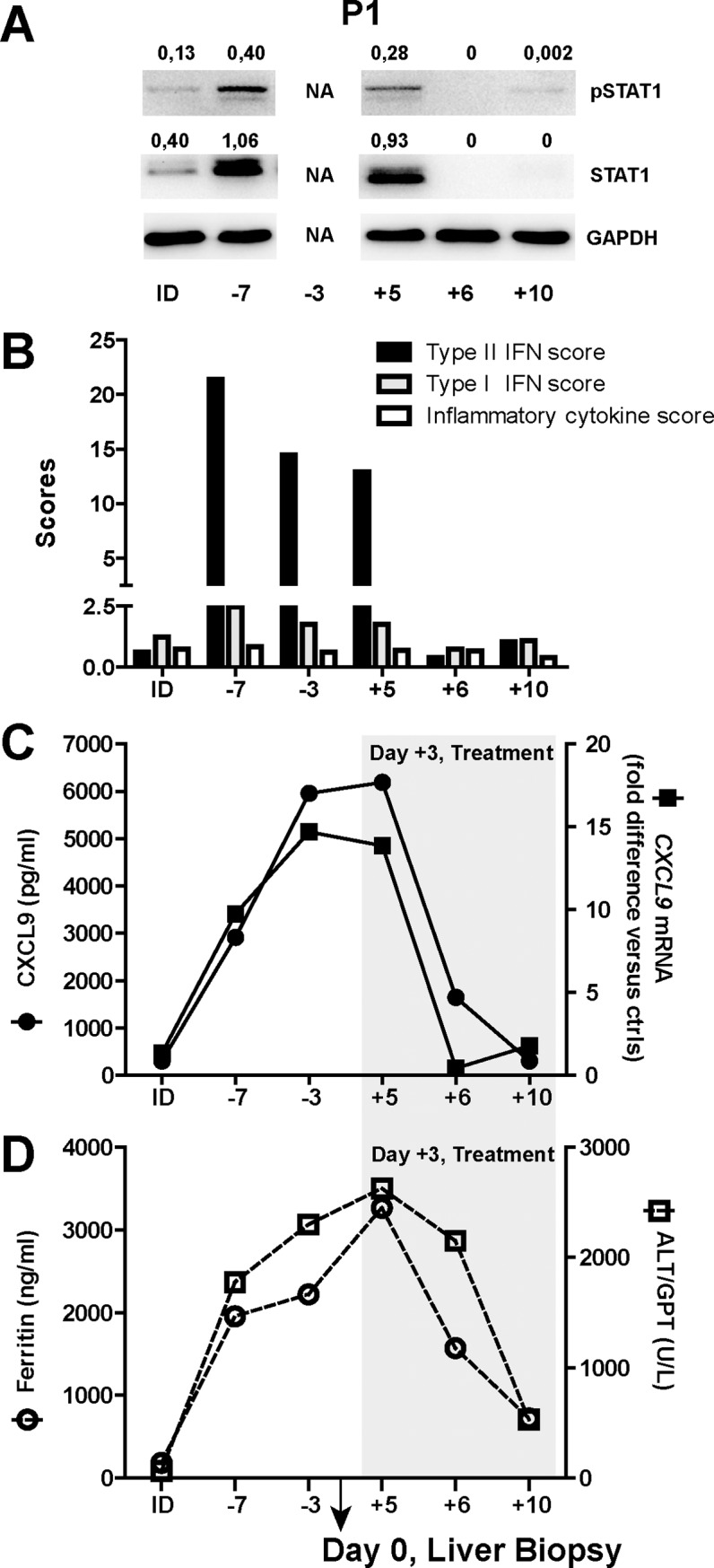
The activation of the IFNγ pathway is up-regulated in PBMCs of P1 collected during the active phase of sHLH. **A)** Tyr(701)-phosphorylated STAT1 and total STAT1 protein levels were assessed by Western Blot analyses in PBMC lysates collected from P1 during the course of the disease (the day of liver biopsy collection was fixed as day 0). A sample collected 6 months before the sHLH episode, while the patient had inactive disease (ID), was also analyzed. pSTAT1/GAPDH and STAT1/GAPDH relative density values are shown above the blots. **B)** The Type II IFN score, the Type I IFN score and the inflammatory cytokine score obtained in PBMCs was calculated. **C)** *CXCL9* mRNA levels in PBMCs and CXCL9 plasma levels have been measured. **D)** Ferritin and ALT/GPT levels during the course of the disease were reported. The area in grey represents the period in which the P1 was under treatment with glucocorticoids. Not available (NA).

We also investigated the longitudinal trends of parameters of IFNγ pathway activation in peripheral blood and their relations with biochemical parameters of disease activity from P1, the only patient of whom longitudinal samples were available. We found a marked increase in the levels of Tyr(701)-phosphorylated and total STAT1 in PBMC protein lysates during the acute phase of the disease, with a marked decrease following initiation of treatment (Fig 6A), accordingly to results showing that addition of dexamethasone to PBMC cultures resulted in a dramatic inhibition of IFN-γ activation of STAT1 [17]. In addition, the mRNA expression levels of *CXCL9* in PBMCs and circulating CXCL9 levels were increased during the active phase of the disease and paralleled the Type II IFN score. In contrast, minor changes in the Type I IFN and the inflammatory cytokine scores were observed during the course of the disease (Fig 6B). Changes in alanine aminotransferase (ALT/GPT) and in ferritin levels were related to the changes in the activation of the IFNγ pathway with initiation of treatment being associated with a decrease in ferritin, liver cytolysis and a decrease in pSTAT1, STAT1, Type II IFN score in PBMCs and circulating CXCL9 levels (Fig 6C and 6D).

## Discussion

We report the marked and selective up-regulation of mRNA levels of *IFNG* and IFNγ-inducible genes in diseased livers of patients with active sHLH. The patients studied had sHLH with predominant liver involvement with no abnormalities in acute phase response blood cell counts and coagulation.

Our observations in the liver from two patient with sHLH and one patient with MAS appear to complement a previous observation from Biliau et al. who reported by immunohistochemistry the presence of IFNγ-producing CD8 positive lymphocytes in affected livers [18]. In this study, we confirm in all the sHLH or MAS patient livers analysed the infiltration from CD8-positive T cells. Interestingly, biopsies from children with acute liver failure of unknown causes are characterized by a dense infiltrate of perforin expressing CD8-positive T cells [19]. Infiltration from CD8-positive T cells has been proposed as a biomarker able to differentiate acute liver failure of unknown causes from acute liver failure with known aetiology (e.g. drugs, metabolic disease, autoimmune process, and infections). It is worth to note that isolated acute liver failure of unknown cause may be a presentation of primary and secondary HLH [9, 20]. The similarities between acute liver failure of unknown aetiology with CD8 infiltrate and the hyperinflammatory state of HLH have been recently underscored [21].

Even if we have not produced evidence confirming that IFNγ is produced by CD8 T cells, altogether, our data and those by Biliau et al. [18] strongly support the conclusion that CD8 cells are the source of IFNγ. Whatever the cellular source, our results demonstrate that events downstream to IFNγ are markedly up-regulated in the liver being consistent with the hypothesis that IFNγ is a major pathogenic mediator of tissue damage. We demonstrate a marked increase in the tissue levels of phosphorylated and total STAT1, a key transcription factor in the signal transduction downstream of the Type II IFN receptor. We report also high expression levels of several genes induced by IFNγ and we derived a Type II IFN score that could be used to evaluate activation of the IFNγ pathway in target organs as well as in peripheral blood (see below), in a manner similar to what is done for Type I score in interferonopathies [14]. Higher expression of the IFNγ-inducible protein IDO and CXCL10 in one lymph node biopsy specimen from one patient with MAS during the active phase of sJIA has been previously reported [22]. In the liver of patients with sHLH and of patient with MAS (even though the latter liver biopsy was collected while she was on glucocorticoids), the marked increase in the expression of IFNγ inducible genes, comprehensively represented by the Type II IFN score, appears to be selective. Indeed, the increase in the expression of Type I IFN inducible genes and of inflammatory cytokine genes, comprehensively represented by the Type I IFN score and by the inflammatory cytokine score, was mild and in the range of that observed in the P4 with autoimmune hepatitis.

Altogether, our results show that a selective and marked activation of the IFNγ pathway is present in the affected livers of patients with sHLH or MAS who do not present systemic symptoms and do not show peripheral blood laboratory features characteristic of HLH. Interestingly, when we studied peripheral blood samples collected during the active phase of the disease, before treatments were initiated, we found that *IFNG* expression was downregulated compared to controls, demonstrating that in these patients IFNγ production is limited to disease target organs. Moreover, only in one patient out of three we found measurable circulating IFNγ levels (Table 1). This is consistent with data from other studies demonstrating a large variability in IFNγ levels (ranging from low picograms to nanograms) in HLH patients [4, 23]. However, even in the absence of systemic symptoms, in the absence of abnormalities in blood cells counts and fibrinogen and in the absence of IFNγ overexpression and overproduction, we found that the mRNA levels of IFNγ inducible genes were markedly higher compared to those observed in healthy controls. It is possible that minor increases in circulating IFNγ levels, possibly spilling out of affected tissues, lead to the activation of the IFNγ downstream pathway. Another, not mutually exclusive, explanation is based on the possibility that circulating cells get in contact with IFNγ being overproduced only in target tissues (in this case liver). Indeed, in mouse models of infection driven HLH and of MAS, overexpression of IFNγ was demonstrated in target tissues [8, 24]. Moreover, in the model of infection driven HLH, by exploiting the ability of an anti-mouse IFNγ monoclonal antibody to capture tissue IFNγ and mobilize it in peripheral blood as an immune complex, a massive increase in detectable circulating IFNγ was observed [24].

In PBMCs longitudinally collected from P1 we found high levels of total STAT1 and phosphorylated STAT1 in samples collected during the active phase of the disease compared to samples collected after the initiation of treatments and during improvement of the disease. In addition, only Type II IFN score, but not the Type I IFN score or the inflammatory cytokine score, changed during the course of the disease, paralleling the increase and the decrease in ferritin and transaminase levels. We also found that the circulating CXCL9 levels reflected the trend of Type II IFN score in PBMCs as well as the trend in levels of ferritin and transaminase. Consistent with our results, recently, transcriptional analysis, performed on whole blood samples from two patients mutated in NLRC4 with MAS collected during flare and after clinical improvement, identified the IFN-γ–associated network as the most enriched [25].

In conclusion, our results provide evidence of selective exaggerated production of IFNγ, and activation of its downstream pathway, in the target organ of patients with HLH with predominant liver involvement. Our data also suggest that evaluation of the IFNγ pathway, as measured by circulating levels of CXCL9 and transcriptional levels of IFNγ inducible genes in PBMCs and evaluation of the Type II IFN score, may be of diagnostic help in patients with suspected HLH who do not fulfil the available diagnostic criteria. It is conceivable that these tests may also help in differentiating HLH with predominant liver involvement from acute hepatitis of other origins. Our findings may also have therapeutic relevance. Indeed, the growing body of evidence pointing to IFNγ as a therapeutic target in primary and secondary HLH, led to clinical trials with emapalumab, an IFNγ neutralizing antibody, in patients with primary HLH or with MAS during sJIA [26, 27]. It is therefore conceivable to hypothesize that IFNγ may be a valuable target also in patients with HLH with prominent/restricted involvement of a single organ.
